# Opportunities and Challenges in Animal Protein Industry Sustainability: The Battle Between Science and Consumer Perception

**DOI:** 10.1093/af/vfaa034

**Published:** 2020-10-30

**Authors:** Judith L Capper

**Affiliations:** Livestock Sustainability Consultancy, Harwell, Oxfordshire, UK

**Keywords:** animal health, carbon footprint, greenhouse gas emissions, meat sustainability, sustainable intensification

**Implications**

- Livestock productivity and efficiency have increased significantly over the past 50 yr, with concurrent reductions in resource use and greenhouse gas emissions.
- Future food demand means that these gains must continue to be achieved through improved genetics, nutrition, husbandry, and adoption of performance-enhancing technologies.
- Future livestock industry sustainability will be enhanced by advances in climate science, use of by-product and novel feeds, and improved animal health and welfare, yet maintaining and improving consumer trust will be paramount.
- A culture of continuous improvement must be adopted to drive forwards sustainable intensification and to communicate the industry’s dedication to improving sustainability.

## The Challenge

The global animal protein industry faces an unprecedented challenge as we enter the third decade of the 21st century. With more than 7 billion people currently inhabiting the planet, our population is predicted to reach 9.6 billion by 2050. Population growth and a rise in per capita income in low-income countries are predicted to increase food demand by 48.6% (FAO, 2017). Yet, this demand must be fulfilled sustainably, and the mechanisms to achieve this are among the most often discussed issues within agriculture given concerns about climate change, resource use, animal welfare, antimicrobial resistance, and the provision of safe, affordable food. Many definitions of “sustainable” exist—for the purposes of this article the author will refer to sustainability as a balance between economic viability, environmental responsibility, and social acceptability.

Future sustainability will depend on livestock producers improving productivity and efficiency so that more meat and milk can be produced using fewer resources, however, consumers increasingly question the methods by which food is produced. The future of the global animal protein industry therefore depends on producers demonstrating their dedication to environmental responsibility while maintaining a positive consumer image of animal agriculture. This “license to operate” should ensure continuing market access for animal proteins and therefore economic viability of livestock production. The challenge is easy to identify, yet far harder to achieve—this article examines the opportunities that will have to be seized by the global livestock industry, using the U.S. industry as an example, to ensure a bright future for animal protein production over the next 30 yr.

## Where Has the Industry Come From?

Environmental responsibility encompasses multiple impacts, yet the focus to date has been on greenhouse gas (**GHG**) emissions and it’s fair to say that both the global and the U.S. livestock industries were unprepared for the extent of governmental, retailer, and consumer interest that arose from the first mention of GHG emissions in the early 21st century. Prior to publication of the FAO (2006) report “Livestock’s Long Shadow,” few were aware of the potential linkages between animal agriculture and climate change, and the FAO’s suggestion (since shown to be inaccurate) that 18% of global anthropogenic GHG emissions were derived from animal agriculture continues to gain significant media coverage.

The considerable progress made by U.S. livestock industries in cutting resource use and GHG emissions over time is a massive achievement for which livestock producers should be applauded. However, much of this progress was a side-benefit of improved productivity and efficiency, rather than an intentional strategy for improving environmental sustainability. For example, Capper (2011) reported that U.S. beef production in 2007 required 19% less feed, 33% less land, and 12% less water and had a 16% reduction in GHG emissions per kilogram of beef compared with production in 1977. These environmental gains were attributed to improved cattle growth rates, slaughter weights and crop yields, rather than the intentional adoption of management practices or systems that would reduce environmental impacts. Similar results were reported by Legesse et al. (2016) for the Canadian beef industry in 1981 compared with 2011—1 kg of beef produced in 2011 required considerably less land and conferred a 14% reduction in total GHG emissions per kilogram of beef. Similarly, within U.S. swine production, a 29% increase in the number of pigs marketed from an improved breeding herd resulted in reductions in feed, land, water, and GHG emissions per kilogram of pork of 67%, 41%, 22%, and 35%, respectively, between 1959 and 2009 (Cady et al., 2013). Additionally, both resource use and GHG emissions per ton of eggs were significantly reduced between 1960 and 2010 as a consequence of improved productivity in U.S. egg systems (Xin et al., 2013). Finally, Capper et al. (2009) reported that a 4-fold increase in milk yield per U.S. dairy cow between 1944 and 2007 reduced feed use by 77%, land use by 90%, water use by 65% and resulted in GHG emissions that were 63% lower per kilogram of milk. A recent follow-up study showed that productivity gains between 2007 and 2017 further reduced land use (20.8%,) water use (30.5%), fuel use (20.2%), and resulted in a 19.2% decrease in GHG emissions per kilogram of milk produced (Capper and Cady, 2019).

A considerable body of evidence exists relating to improving animal productivity on resource use and GHG emissions. However, is a business-as-usual, productivity-focused approach enough to carry the animal protein industry into a sustainable future? Considerable media speculation exists as to whether livestock has reached peak productivity, beyond which animal health or welfare may be impaired. The high feed conversion efficiencies, reproductive performance, and growth rates of swine and poultry suggest that a maximum may be within sight; however, in ruminant livestock, there is evidence that productivity has yet to peak. For example, the world record dairy cow, kept in a 360 cow-herd in Wisconsin, USA, produced 35,457 kg of milk in a single lactation (234% more than the average U.S. cow in 2019), suggesting that the linear increase in national average milk yields may continue into the future, with further environmental gains possible. Productivity gains may also be augmented in beef and dairy cattle by the adoption of performance-enhancing technologies. Such technologies include ionophores, orally active or in-feed hormones, implantable and injectable hormones, and β-adrenergic agonists, and have been used for decades within livestock production, improving efficiency, and therefore enhancing sustainability, via the “dilution of maintenance” effect (Johnson et al., 2013). Although performance-enhancing technology adoption varies across the global livestock industry, these technologies have clear positive impacts on economic viability (improved producer income) and environmental sustainability (reduced resource use and GHG emissions per unit of animal protein produced) detailed across multiple studies (Capper et al., 2008; Capper, 2012; Capper and Hayes, 2012; Webb et al., 2017). It seems obvious that these technologies should be part of the suite of tools required to improve sustainability, although regulatory barriers may exist that prevent their wholescale adoption (Dilger et al., 2016). However, although we celebrate efficiency and technology in many industries, these words sometimes appear as lightning rods within sustainability discussions.

## In Science We Trust?

Consumer mistrust of technology in food production is not a new construct—it was seen at the introduction of pasteurized milk in the 1890s, artificial colors and flavorings in the 1960s and irradiated meat in 1990s (Freidberg, 2009). Consumers often show an inherent bias toward foods or systems that they perceive to be better from an animal welfare, environmental impact, or human health perspective. However, as these perceptions are often emotional rather than evidence-based, choosing the philosophically “better” option may lead to negative trade-offs, especially with regards to GHG emissions or resource use.

**Figure 1.**
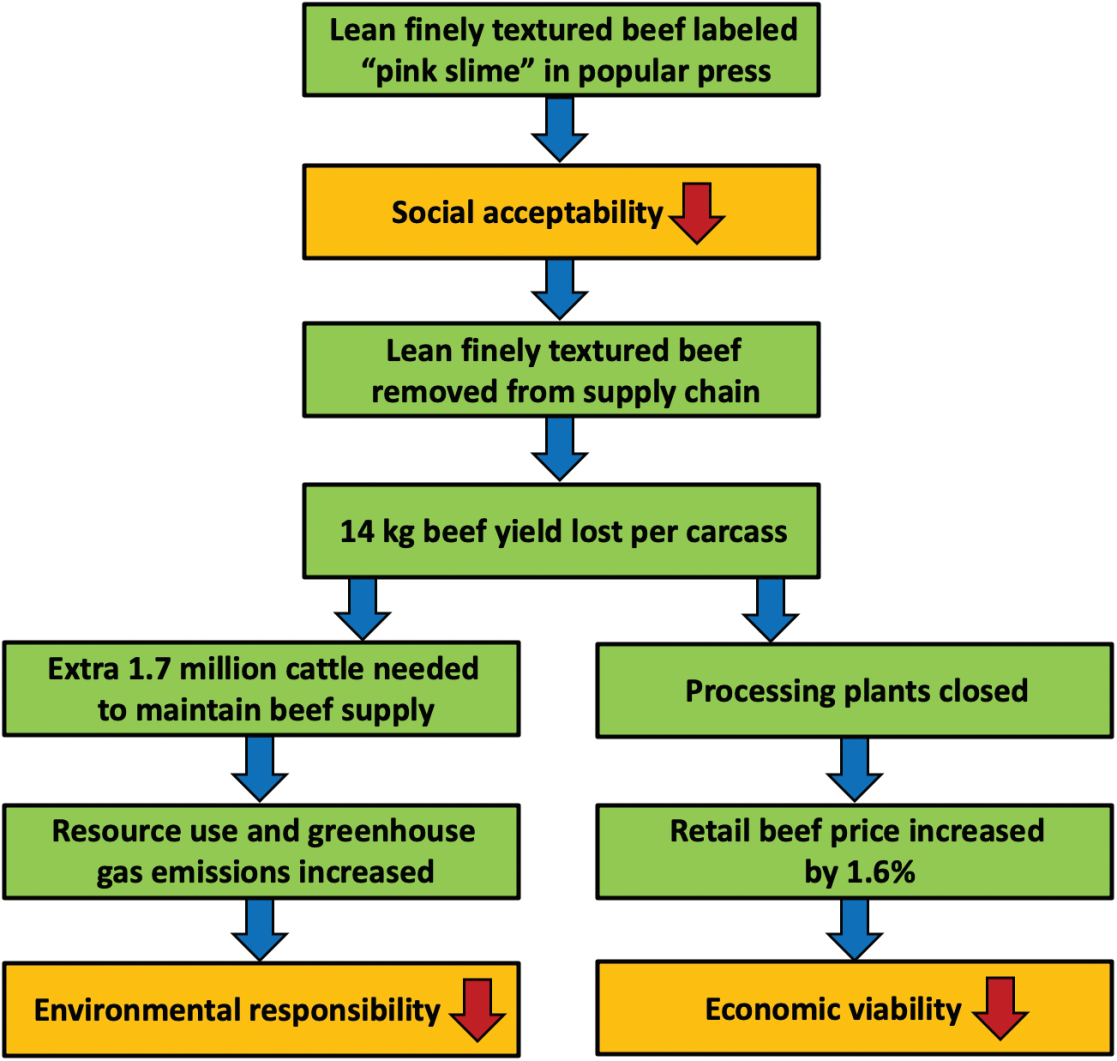
The impact of lean finely textured beef removal on U.S. beef industry sustainability.

Perhaps the best recent example of this technology conflict, was the 2012 controversy relating to lean, finely textured beef. Harvesting lean, finely textured beef allowed processors to gain ~14 kg extra meat from each beef carcass. Yet, after significant negative publicity in which the product was labeled “pink slime,” the lean, finely textured beef component was withdrawn from the ground beef offering in many U.S. grocery stores. Resulting economic losses increased the retail price of beef by 1.6% (Hayes and Otto, 2012) and necessitated an extra 1.7 million head of cattle in the national beef herd (author’s calculation) to maintain beef production, with concomitant increases in resource use and GHG emissions (Figure 1).

**Figure 2.**
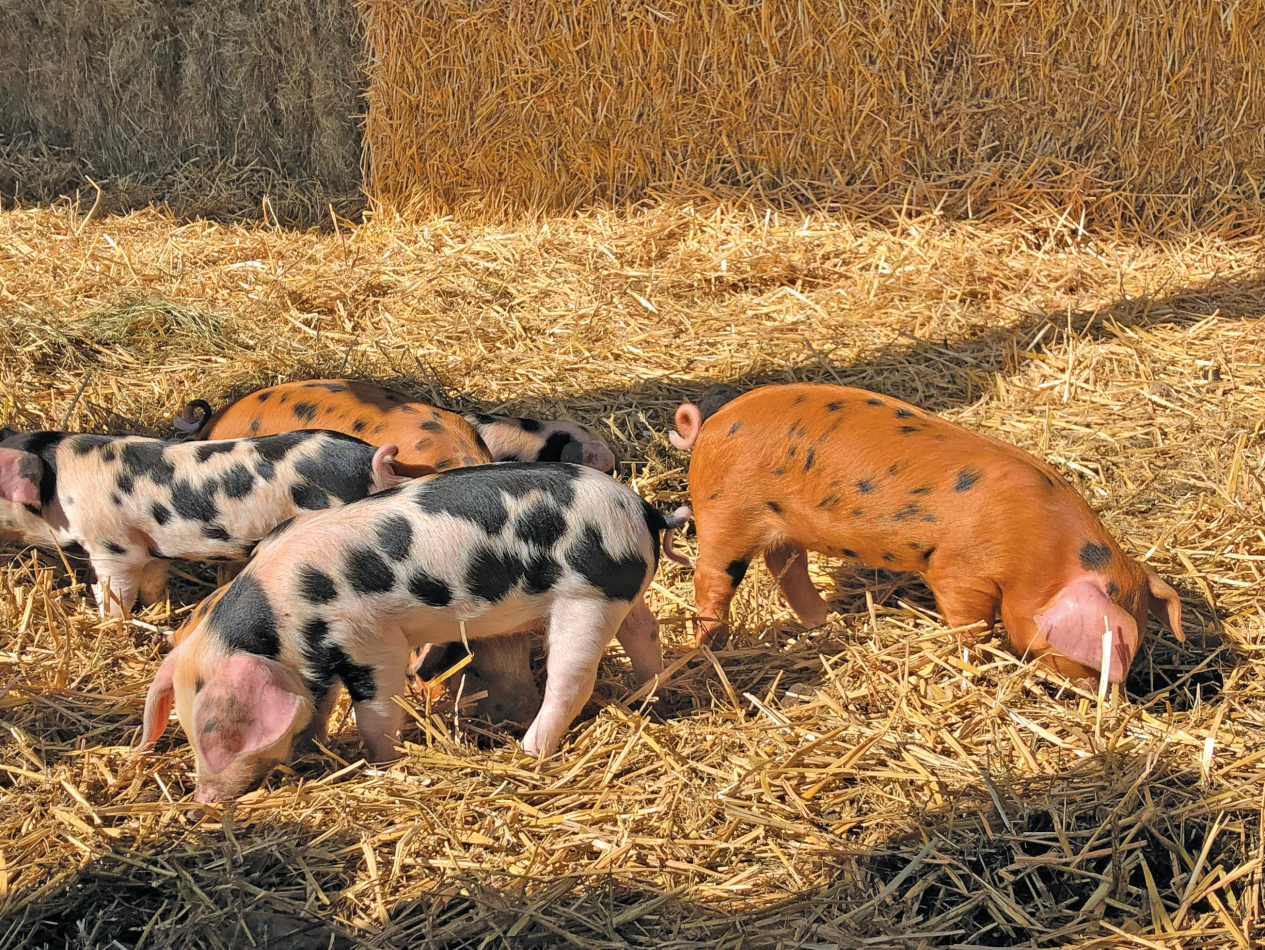
Piglets in a deep-bedded housing system.

We are all consumers and often view food issues through our rose-tinted views of traditional food production, casting a flattering light on extensive systems and assuming that these are more environmentally friendly than intensive systems, yet it is seldom that simple. Both Capper (2012) and Pelletier (2010b) reported increased land use per kilogram beef in grass-fed beef systems, and Capper (2012) further reported a 302% increase in water use and 68% increase in GHG emissions per kilogram of beef compared with conventional beef feedlot systems. Similarly, Pelletier (2010a) found that conventional U.S. swine production systems had lower energy use, GHG emissions and ecological footprints compared to deep-bedded “niche” sow systems (Figure 2).

When purchasing food, an attitude-behavior gap exists between how we would wish to be perceived as citizens (“I care about environmental sustainability”) and how we actually behave in the grocery store (“I choose food based on price, not carbon footprint”), therefore commonly reported concerns about climate change, animal welfare or veterinary medicine use may not be represented by the foods found within our shopping cart. Nonetheless, if other sustainability attributes are valued above environmental responsibility, or if philosophical concerns outweigh evidence-based decisions, consumers may move toward, for example, buying meat from organic systems in the belief that these systems must have lower GHG emissions, or buying milk from pasture-based dairy farms on the basis that cattle have to graze to be “happy.” Future livestock industry sustainability therefore depends on successfully communicating the relative importance of environmental responsibility and both quantifying and qualifying the sustainability attributes of differing production systems.

Continuing to improve productivity on a global scale should enhance the sustainability of future livestock production. This will be particularly crucial in underdeveloped regions where livestock currently exhibit low productivity, although changes to breeds, practices, or production systems must consider the opportunities and limitations of climate, resources, cultural factors, markets, and infrastructure. For example, supplying high-yielding Holstein cattle to regions with high temperatures and little shade or housing, or dairy goats to regions where drinking goat milk is culturally undesirable is unlikely to result in resilient, sustainable systems. Although further production intensification will be crucial, the previous business-as-usual model will have to be refined to provide sustainable intensification. The Foresight (2011) definition: “an intensive food production system that encompasses methods and practices to reduce both chemical inputs and negative environmental impacts” will provide a solid foundation, yet it must be broadened to include other economic, social, and animal health and welfare issues. Practices to improve sustainability must be targeted at the individual system or farm level and cannot be prescribed as “one-size-fits-all,” therefore although the goal of future livestock production will be to achieve industry sustainability, it will be achieved through a myriad of different initiatives.

## What Else Must the Industry Do?

The animal protein industry faces a number of challenges moving forward—many of which are beyond the scope of this paper, yet there are three clear issues that need to be addressed immediately: (1) changing the accounting systems for GHG emissions, (2) embracing non-human-edible feedstuffs, and (3) prioritizing animal health and welfare.

The current industry focus on GHG emissions as the principal measure of environmental sustainability is understandable—climate change certainly appears to be the most discussed, and potentially most dangerous environmental issue currently on the global radar. Yet this focus is somewhat myopic as it does not consider the potential negative consequences of essentially having a single environmental metric. If a retailer decides to reduce the GHG emissions from their supply chain by only sourcing from those with a carbon footprint lower than a specific cut-off point, this may unintentionally restrict the supply pool to those operations that, for example, have less biodiversity, higher levels of soil erosion or more water pollution, or that include practices that some consumers may consider to be undesirable (e.g., continuous housing).

**Figure 3.**
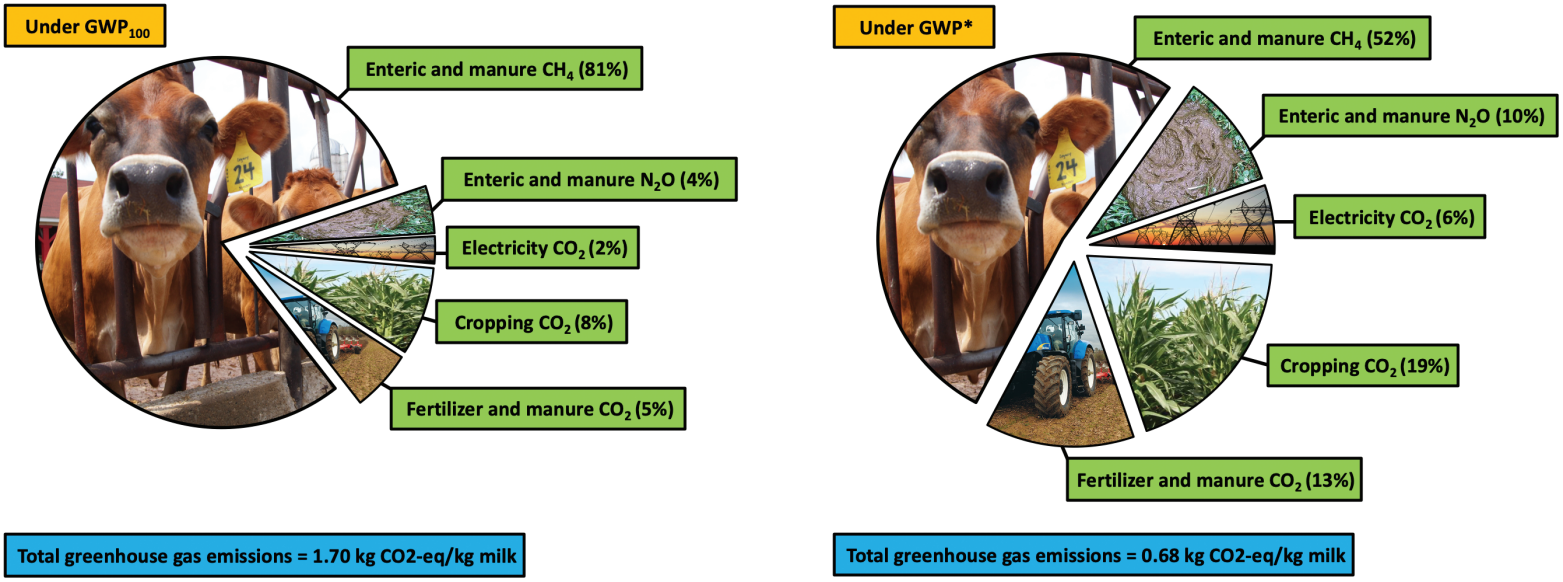
Greenhouse gas emissions per unit of U.S. milk under GWP100 and GWP* (adapted from Capper and Cady, 2019).

The ways in which GHG emissions are quantified and used to compare livestock systems may also be revolutionized in the near future by new research emerging from Allen et al. (2018) at Oxford University. Methane, the primary GHG emitted from livestock systems, was previously thought to accumulate in the atmosphere indefinitely. However, a new GHG metric, GWP*, encompasses the fact that methane breaks down over time, reducing its relative ability to cause global warming. This would potentially cut total GHG emissions per kilogram of U.S. milk or beef by ~60% (Figure 3, author’s calculation). This new metric would have the greatest impact on systems whose total GHG emissions have a relatively higher proportion of methane, i.e., more extensive, pasture-based systems, therefore might reduce the variation in GHG emissions between intensive and extensive systems.

At the time of writing, GWP* has not been unilaterally adopted by the international bodies assessing climate change and GHG emissions, therefore the global ruminant industry would benefit from lobbying to effect this change. The potential adoption of GWP* must not be seen as an environmental “get out of jail free” card, with the associated assumption that the ruminant industries need not reduce environmental impacts in future. If GWP* is adopted, the current focus on methane may be redirected toward carbon dioxide and nitrous oxide and therefore more attention paid to fossil fuel combustion, crop production, and manure management (Figure 3).

Public unease regarding the use of human-edible grains and oilseeds to feed livestock is likely to continue, therefore sustainable intensification must include using non-human-edible by-product feeds, e.g., citrus pulp or distillers grains. Metrics for feed conversion efficiency (e.g., 27.5 kg of feed per kg of grass-fed beef) should be revised to account for whether humans can or will consume feed ingredients and food outputs (e.g., 0.9 kg human-edible protein input per kg human-edible protein output in beef), as suggested by Wilkinson (2011). This would allow the “feed vs. food” debate and the potential for novel livestock feeds (e.g., insect protein) to be contextualized.

Animal health and welfare must be priorities for livestock systems going forward. The impacts of disease on productivity are well detailed, and it should be no surprise that animals suffering from clinical or subclinical disease perform less well than their healthy herd or flock mates. Globally, 20% of animal protein is lost due to animal diseases (OIE, 2015), many of which have effective preventative or curative treatments already developed, but not adopted worldwide due to economic, infrastructure, regulatory, or political disconnects. However, remarkably little contemporary data exist upon the sustainability impacts of livestock disease, even for diseases that can have catastrophic effects in the event of an outbreak (e.g., Johne’s disease in cattle or porcine respiratory and reproductive syndrome in pigs). This means the majority of producers, processors, and retailers are unable to understand and quantify the relative economic and environmental cost:benefit ratio of animal health practices and to therefore make informed management, sourcing, and price decisions.

Spain et al. (2018) reported that 78% of surveyed consumers were concerned about the welfare of U.S. livestock and believed that animal welfare should be audited by an objective third party, with 70% paying attention to labeling information indicating how animals were raised and 57% being likely to choose a restaurant because it offered welfare-certified products. The impact of consumer preferences should not be underestimated—future livestock systems will either have to demonstrate that production intensification can be synonymous with good health and welfare, or amend systems accordingly, such that an acceptable middle ground can be found.

Reducing antimicrobial resistance will also be a key concern in maintaining social acceptability of future livestock systems. Action must be taken by livestock producers to reduce, replace, and refine antimicrobials that have equivalents in human medicine, yet Cervantes (2015) suggested that antimicrobial-free poultry production would be unsustainable in U.S. systems due to negative effects upon bird health. Furthermore, Karavolias et al. (2018) concluded that removing antimicrobials from poultry production would increase both the risk and the severity of specific diseases, therefore this might be a considerable challenge in current intensive production systems. However, one British veterinary practice reported that, over a 5-yr period, use of highest-priority critically important antimicrobials could be cut by 91%, with no actual or perceived evidence of declining herd health or poorer treatment outcome (Tisdall et al., 2017). Future livestock systems must implement best practices to improve biosecurity, disease surveillance, resistance monitoring and livestock husbandry, and adopt vaccines where possible. However, this presupposes that consumers will still be purchasing milk, meat, and eggs in future—is this a logical supposition, or are we trying too hard to convince ourselves?

## Is the Future Bright for Animal Protein Industry?

We may not have reached the dystopian states imagined in Huxley’s (1932) Brave New World or the 1973 film Soylent Green, yet our culture and society has changed considerably over the past century. In 1919, the average American spent 38.2% of their income on food, a kilogram of pork chops cost $0.86 (Chao and Utgoff, 2006), and people would go to considerable lengths to ensure that a cut of beef or pork would last for several days rather than being consumed in a single meal. By contrast, in 2019, only 9.7% of income was spent on food, 48% of that spend was consumed outside the home (USDA Economic Research Service, 2020), and meat consumption per capita had almost doubled. While it is tempting to suggest that everything was better in the good old days, as per the often suggested “Don’t eat anything your great-grandmother wouldn’t recognize as food” (Pollan, 2009). this does not necessarily result in a healthier or better diet. Consuming diets similar to those enjoyed over 50 yr ago would indeed eliminate some ultra-processed foods, including many of the plant-based alternatives to animal proteins, but would also remove many food additives that improve food quality, shelf-life, or safety. However, it is clear that the way that we choose, buy, and consume food will change in future.

Images of grocery store shelves stripped bare of milk, meat, and eggs during the recent COVID-19 pandemic demonstrated that, although many consumers may cite an intention to reduce animal protein consumption, this may be a somewhat artificial construct, only practiced when food is assumed to be plentiful. Under conditions where food availability may be restricted, consumers appear to demand milk, meat, and eggs as essential staples. This is reinforced by the anecdotal claim that, globally, cheese is the food most-often stolen food, a dubious honor based on cheese’s nutritional value, portability, and popularity.

The popularity of campaigns such as Veganuary often leads to the supposition that everybody is likely to adopt a vegan lifestyle in the near future, although vegans only comprise a small proportion of the U.S. population, at ~3% of the total. The range of “vegan friendly” foods available in grocery stores has increased exponentially over the past three decades. However, we simply do not know whether plant-based foods are replacing animal proteins or whether consumers are simply enjoying dietary diversity and using new foods to augment their existing diet. However, in future, we are unlikely to follow the same “meat and potatoes” model for food consumption as enjoyed in the past, nor to transition to a wholly vegetarian or vegan diet, but may adopt a more flexitarian (intentionally choosing to eliminate meat from a proportion of meals) approach. This will not occur overnight, but may result from a series of incremental behavioral changes over time in the same way that, for example, recycling paper and plastics or using reusable shopping totes have moved from niche practices only seen in urban neighborhoods, into the mainstream. Rather than seeing animal proteins as staple foods with purchasing decisions primarily placed on price, an “eat less but better” approach may be adopted. This may be more likely if taxes are applied to animal proteins in an attempt to reduce consumption, and would offer marketing opportunities based on improved animal welfare, environmental impacts, or nutritional quality. In future, attributes that are not specifically valued within the current retail price may become be an inherent cost of gaining a social license to operate, therefore it will be necessary to measure, benchmark and improve animal welfare, GHG emissions, AM use, and other parameters.

**Figure 4.**
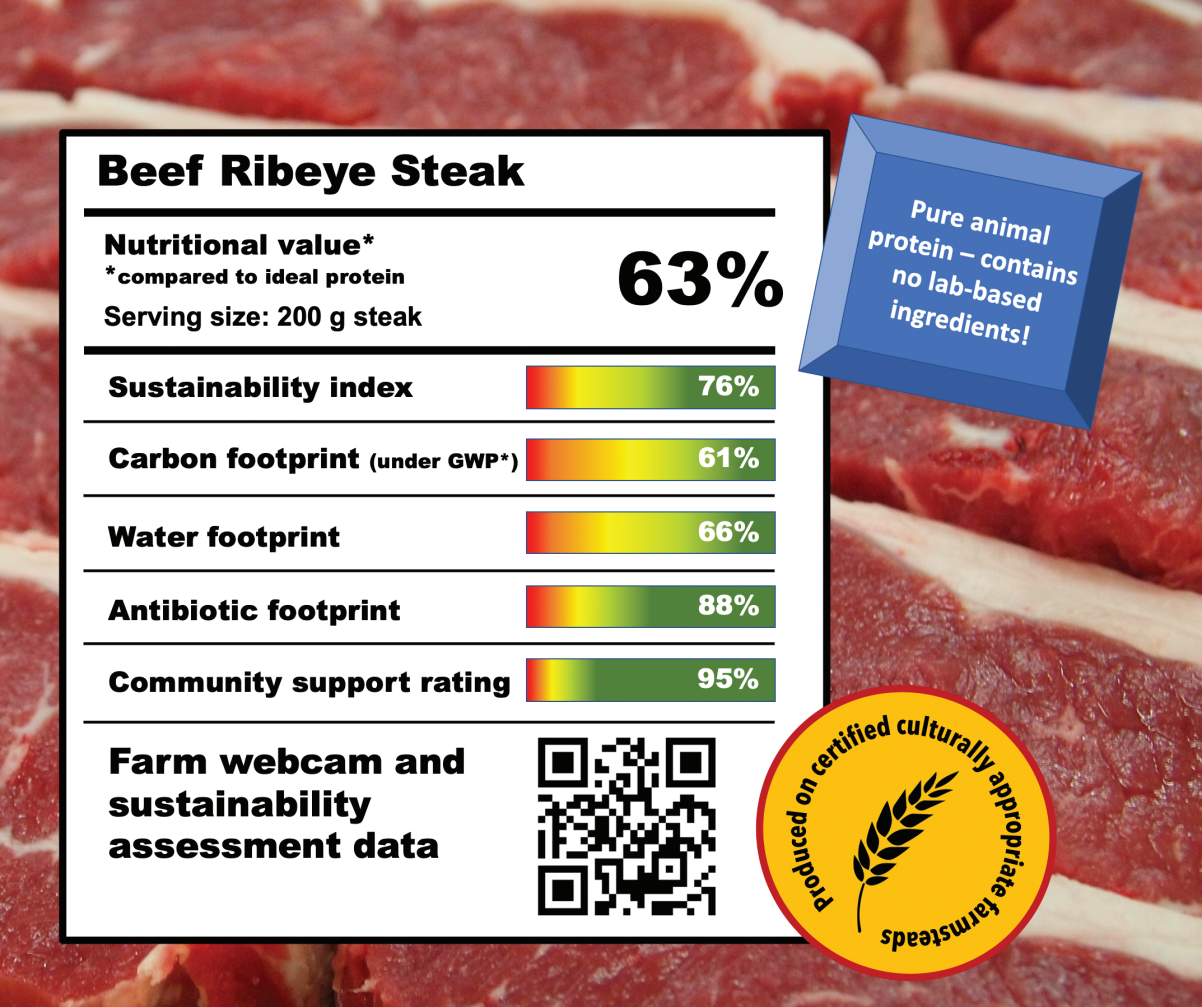
Is this what meat labels will look like in the future?

Historical projections of future lifestyles suggested that by 2020, we might all drive flying cars, be served by robotic butlers and have dispensed with food and drink in favor of nutrient pills that would fulfill our daily needs without wasting time or resources preparing and eating meals. We have seen a rise in the popularity of prepackaged, reheatable meals over the years, and “lab-based” meat is often promoted in the media. However, the increasing tendency to regard food as an experience rather than a package of nutrients (as evidenced by the popularity of food photos on social media site Instagram) suggests that although technology will play a key role in future food production, it will enhance rather than eliminate animal protein consumption. Beyond the on-farm technologies discussed earlier in this paper, at the processing and the retail level, technology may focus on quality, safety, and education, including interactive logos or QR codes (Figure 4) that instantly provide information on how animals were raised, farming systems, and other metrics of interest to fulfill consumers’ hunger for knowledge.

## Conclusions

Ultimately, maintaining and improving consumer trust will be key to maintaining the social acceptability, and therefore overall sustainability of future meat and milk production, which will also be contingent upon better communication throughout the food chain. Without a social license to operate granted by consumers, animal proteins may be safe, affordable, have minimal impact on the environment and offer tremendous standards of animal welfare, but will be operating without a solid market foundation. Both the United States and the global animal protein industries must therefore take the opportunity to open communication channels that will educate and excite the younger generation that will be making purchasing decisions within 10 yr. It is crucial to ensure that the environmental and economic benefits of livestock production are understood by the public, such that threats to system resilience (e.g., animal welfare exposés or claims about negative environmental impacts) are negated, because dedication to improving sustainability has been clearly outlined, demonstrated, and communicated. The challenge to the industry is to adopt a culture of continuous improvement in driving forward sustainable intensification, encompassing improved health for animals, people, and the planet; to adopt both existing and new technologies; and to communicate dedication to improving sustainability to all food stakeholders. The future is not simply bright—it is dazzling.

*Conflict of interest statement*. None declared.

## About the Author

Judith (Jude) L. Capper, PhD, is an independent Livestock Sustainability Consultant based in the UK. Jude’s research focuses on modelling the impacts of animal productivity and health (specifically dairy and beef), on livestock system sustainability. Her professional goal is to communicate factors affecting livestock sustainability to enhance the knowledge and understanding of food production stakeholders and, to this end, she has an active social media presence, spending a considerable amount of time de-bunking the more commonly heard myths relating to livestock production. In recognition of this work, Jude was awarded Associate Membership of the Royal Agricultural Society in 2018.
